# Application of optimal band-limited control protocols to quantum noise sensing

**DOI:** 10.1038/s41467-017-02298-2

**Published:** 2017-12-19

**Authors:** V. M. Frey, S. Mavadia, L. M. Norris, W. de Ferranti, D. Lucarelli, L. Viola, M. J. Biercuk

**Affiliations:** 1 ARC Centre for Engineered Quantum Systems, School of Physics, The University of Sydney, Sydney, NSW 2006 Australia; 2 National Measurement Institute, West Lindfield, NSW 2070 Australia; 3 Department of Physics and Astronomy, Dartmouth College, 6127 Wilder Laboratory, Hanover, NH 03755 USA; 4 Johns Hopkins University, Applied Physics Laboratory, 11100 Johns Hopkins Road, Laurel, MD 20723 USA

## Abstract

Essential to the functionality of qubit-based sensors are control protocols, which shape their response in frequency space. However, in common control routines out-of-band spectral leakage complicates interpretation of the sensor’s signal. In this work, we leverage discrete prolate spheroidal sequences (a.k.a. Slepian sequences) to synthesize provably optimal narrowband controls ideally suited to spectral estimation of a qubit’s noisy environment. Experiments with trapped ions demonstrate how spectral leakage may be reduced by orders of magnitude over conventional controls when a near resonant driving field is modulated by Slepians, and how the desired narrowband sensitivity may be tuned using concepts from RF engineering. We demonstrate that classical multitaper techniques for spectral analysis can be ported to the quantum domain and combined with Bayesian estimation tools to experimentally reconstruct complex noise spectra. We then deploy these techniques to identify previously immeasurable frequency-resolved amplitude noise in our qubit’s microwave synthesis chain.

## Abstract (web summary)

Control of qubits’ frequency response by dynamical decoupling is usually vexed by control’s out-of-band harmonics, a problem known in metrology as “spectral leakage”. Here, the authors reduce this problem by orders of magnitude exploiting discrete prolate spheroidal sequences to control a trapped-ion qubit.

## Introduction

**Fig. 1:**
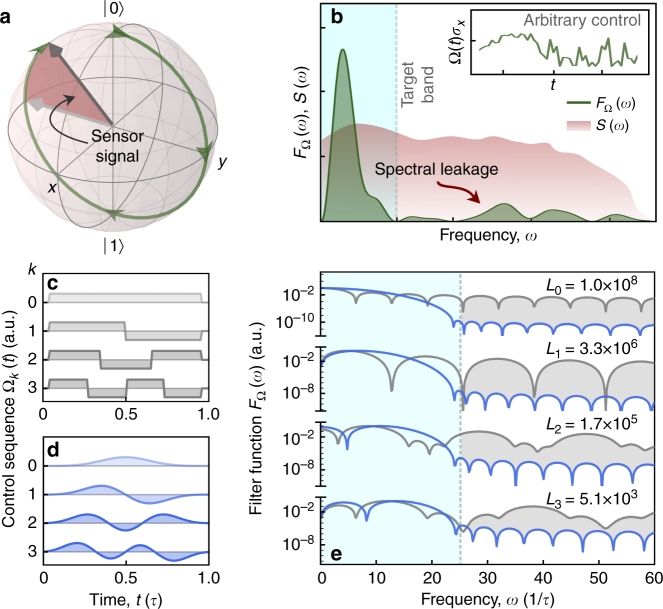
Illustration of frequency response of time–domain control protocols. **a** Sensor signal for a driven qubit is derived from the spectral overlap of the noise and control, producing a detectable rotation error. **b** Sensor spectral response for a given control protocol (inset) may show leakage outside of the desired target band, which makes an undesired contribution to the signal in **a**. **c**, **d** Sample control sequences for flat-top echo protocols and their DPSS-modulated counterparts. Orders shown here: *k* = 0, 1, 2, 3, and *NW* = 4. Note that *k* is equal to the number of zero crossings in the time domain and, by construction, odd-order pulses, with zero net rotation, have no DC susceptibility. **e** *F*_Ω_(*ω*) capturing the frequency response of the control envelopes in **c**, **d**. The dotted line indicates the boundary of the target band along the positive frequency axis, *ω*_*B*_ = 2*πW*/Δ*t*, where *τ* = *N*Δ*t* is the pulse duration. Inferior spectral concentration of flat-top (FT) pulses, manifested as spectral weight beyond *ω*_*B*_, is highlighted with shading. Out-of-band power relative to *k*th order DPSS-modulated pulses is calculated as $L_k \equiv \left( {1 - \lambda _k^{{\mathrm{FT}}}} \right){\mathrm{/}}\left( {1 - \lambda _k^{{\mathrm{DPSS}}}} \right)$, where $\lambda _k^\ell = {\int}_0^{\omega _{{B}}} {\mathrm{d}}\omega F_\Omega ^\ell (\omega ){\mathrm{/}}{\int}_0^\infty {\mathrm{d}}\omega F_\Omega ^\ell (\omega )$, for the appropriate *k*th order filter functions

Industrial, metrological, and medical applications provide a strong pull for advanced nanoscale sensing techniques^1^, exploiting the exquisite sensitivity of quantum coherent systems to their environments^2–4^. Qubits naturally exhibit broadband coupling to their environments, but the application of a temporally modulated driving field can alter their frequency response in a desired way. For instance, application of modulation which periodically flips the qubit’s state has allowed for a narrowband spectral response^5^, which may be tuned by adjusting the interpulse spacing or extending the sequence duration. This general approach to “dynamical decoupling noise spectroscopy” has seen broad adoption in quantum information^6–11^, as well as in nanoscale diamond sensors for biomedical and physics-based applications^12–15^. However, control implemented in this form suffers from the significant complication of spectral leakage, where signals outside of a target sensing frequency band can contribute to the sensor’s response (Fig. 1b), and if not properly accounted for, can lead to bias when estimating the spectral density of a signal from experimental data^16,17^.

In an ideal scenario, for frequency domain spectral estimation applications, the chosen control protocol would be sensitive only within a user-determined band. Pulsed dynamical decoupling is often employed because the leading component of the filter transfer function describing the modulated sensor’s performance is narrowly peaked^5,7^. Examination of the control propagator describing the time–domain response of a qubit subject to this control, however, reveals that the effective square-waveform of the control propagator (Fig. 1c) leads to the appearance of an infinite chain of harmonics in the Fourier domain (Fig. 1e). These out-of-band harmonics can then contribute bias in noise spectroscopy protocols.

The problem of spectral leakage is well known in classical signal processing and has led to the development of time–bandwidth-optimized functions for use in spectral estimation. The discrete prolate spheroidal sequences (DPSS)^18^ are an orthogonal set of discrete time functions that maximize a signal’s energy within a predefined frequency band (Fig. 1d)^19^. The DPSS form the basis of the multitaper method of spectral analysis^20^, which is employed in estimation problems across a wide range of physical, computational, and biomedical disciplines^21,22^. Additionally, DPSS have also been suggested in magnetic resonance imaging to avoid out-of-band excitation (so-called Gibbs artifact^23^) and have recently enabled the design of optimal control algorithms for unitary quantum dynamics, that incorporate bandwidth constraints^24^. This strong base of demonstrations motivates our use of DPSS-modulated pulses for quantum sensing.

In this work, we adapt DPSS functions to the problem of spectral leakage in quantum control for qubit-based sensors. We introduce the concept of continuously amplitude-modulated control waveforms defined by DPSS functions as effective window functions, and we demonstrate that such controls afford suppression of spectral leakage in the quantum setting. Experiments with trapped atomic ions are used to reconstruct the filter transfer functions and show orders of magnitude improvement in suppression of out-of-band signals relative to conventional square waveforms (e.g., pulsed dynamical decoupling or instantaneous phase-flips under driven rotary echo^25,26^). We then present a series of RF engineering-inspired techniques to shift the band center of the control’s frequency response and combine this with tomographic measurements to allow disambiguation of sensor response in a cluttered background. Two techniques for spectral reconstruction are presented, both inspired by the original classical multitaper method, and we compare their performance for experimentally reconstructed spectra. Experimental results show that our new non-inverting quantum multitaper performs similarly to more computationally intensive Bayesian estimation routines, at the expense of frequency resolution in the reconstruction. Finally, we employ these techniques to characterize otherwise inaccessible properties of our qubit drive system and provide frequency-resolved characterization of system noise and non-linearities with calibrated sensitivity to 0.001 dB.

## Results

### The DPSS functions

For a time–domain sequence consisting of *N* elements, characterized by sampling interval Δ*t*, and half-bandwidth parameter *W* ∈ (0, 1/2), the DPSS may be defined as real solutions to the eigenvalue problem

1 $$\mathop {\sum}\limits_{m = 0}^{N - 1} \frac{{{\mathrm{sin}}\,2\pi W(n - m)}}{{\pi (n - m)}}v_m^{(k)}(N,W) = \lambda _k(N,W)v_n^{(k)}(N,W),$$

where $v_m^{(k)}(N,W)$ is the *n*th element of the *k*th order DPSS for *k*, *n* ∈ {0, 1, …, *N* − 1}. The discrete Fourier transform of $v_m^{(k)}(N,W)$ into the (angular) frequency domain [*−π*/Δ*t*, *π*/Δ*t*] is the discrete prolate spheroidal wavefunction, *U* ^(*k*)^(*N*, *W*;*ω*), which is spectrally concentrated in [*−ω*_*B*_, *ω*_*B*_] ≡ [−2*πW*/Δ*t*, 2*πW*/Δ*t*].

The eigenvalue *λ*_*k*_(*N*, *W*) directly quantifies the spectral concentration of *U* ^(*k*)^(*N*, *W*;*ω*), i.e., the fraction of spectral power within the target band compared to the total spectral power, as $\lambda _k(N,W) = \frac{{{\int}_{ - \omega _B}^{\omega _B} {\rm d}\omega U^{(k)}(N,W;\omega )^2}}{{{\int}_{ - \pi /{\mathrm{\Delta }}t}^{\pi /{\mathrm{\Delta }}t} {\rm d}\omega U^{(k)}(N,W;\omega )^2}}.$ DPSS are optimal in the sense that, among all sequences with fixed *N* and *W*, they are the ones that maximize the above ratio. Spectral concentration is highest for *k* = 0, and decreases with increasing *k*; the DPSS of order $k < 2\left\lfloor {NW} \right\rfloor - 1$ have ≥70% of their spectral weight within the target band (Fig. 1e; Supplementary Table 1). Physically, the time–bandwidth product *NW* controls the fraction of the desired pulse energy within the pulse time *τ* = *N*Δ*t*. Increasing *NW* enables concentration to be maintained for higher *k*, but this has the disadvantage of extending the target band in the frequency domain. In practice, setting *NW* = *k* + 1 is usually a satisfactory compromise between these factors.

### DPSS filter transfer function reconstruction

To characterize the frequency response of a qubit-based sensor undergoing an arbitrary control protocol in the presence of multi-axis classical noise, we rely on the filter transfer function formalism^5,27–31^, which we also outline in the Supplementary Note 4. The qubit sensor’s response to its environment under the application of control is given approximately by the average measured fidelity of the operation, denoted here as ${\cal F}_{{\mathrm{av}}}$. This is captured, in the weak-noise limit (Methods), by the spectral overlap of the noise power spectral density in multiple quadratures, *S*_*i*_(*ω*), with a transfer function describing the control, *F*_*i*_(*ω*), as ${\cal F}_{{\mathrm{av}}} \approx 1 - {\mathrm{exp}}\left[ { - \pi ^{ - 1}\mathop {\sum}\nolimits_{i = \Omega ,z} {\int} {\mathrm{d}}\omega F_i(\omega )S_i(\omega )} \right].$ Here, the sum is taken over noise contributions in the amplitude quadrature, proportional to the applied control, i.e., the qubit Rabi frequency ∝Ω*σ*_*x*_, and in the dephasing quadrature, ∝*σ*_*z*_. The presence of a signal in the sensor’s target band, defined by the applied control modulation, will be manifested as a reduction in the fidelity of the operation implemented. The latter may be the identity, for odd *k*, or, in general, another non-trivial quantum state transformation (Fig. 1a).

Analytic calculation of *F*_Ω_(*ω*) both for flat-top modulation (commonly associated with dynamical decoupling protocols and here a rotary spin echo, Fig. 1c) and for piecewise-constant modulation defined by the DPSS-modulated pulses, reveals the superior spectral concentration of the latter (Fig. 1e). While the main lobe of *F*_Ω_(*ω*) is broader inside the target band (blue shading) for DPSS modulation as compared to the rotary spin echo, leakage outside the target band is significantly suppressed. For the rotary spin echo, spectral leakage increases out-of-band sensitivity by 30–80 dB relative to the DPSS, quantified by the value *L*_*k*_ (Fig. 1e).

We perform experiments to directly test the spectral response of a driven qubit-based sensor using trapped ^171^Yb^+^ ions, where the qubit is realized through the hyperfine splitting of the ^1^S_1/2_ ground state with a transition frequency ~12.6 GHz. We can modulate the amplitude and phase of the driving microwave field arbitrarily using a vector signal generator, providing full control of the qubit state on the Bloch sphere. We employ projective measurements of the qubit state in the *z*-basis and average over experiments to identify deviations from ideal control operations, which constitute our signal of interest, *P*(↑_*z*_). Details of the experimental system appear in refs. ^32,33^ and in the Supplementary Note 1.

**Fig. 2:**
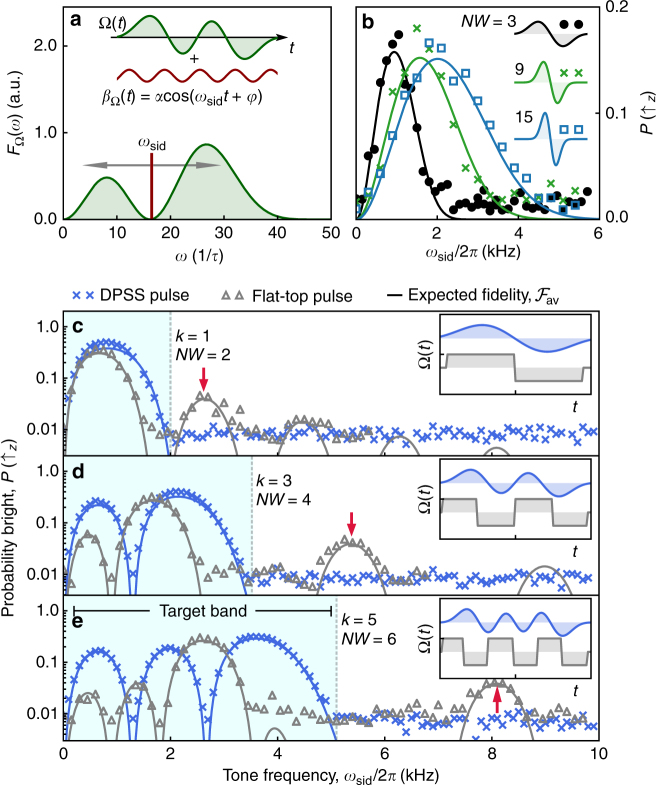
Spectral reconstruction of control filter transfer functions. **a** A single-frequency perturbation, *β*_Ω_(*t*) is applied to the control envelope Ω(*t*), which drives rotations about *x*. Measuring operational fidelity (see main text) for each system identification frequency, *ω*_sid_, allows frequency-resolved reconstruction of *F*_Ω_(*ω*). **b** Experimental reconstruction of DPSS filter functions for *k* = 1 and varying *NW*. Solid lines show the analytic ${\cal F}_{{\mathrm{av}}}$ calculated based on *F*_Ω_(*ω*). Control envelopes (shown schematically as insets) have a duration of 1.1 ms with area normalized to *π* prior to sid perturbation, and measurements are averaged over 10 linearly sampled *φ* ∈ [0, 2*π*], with *α* = 0.5. Each phase realization is repeated 50 times to reduce the influence of photon shot noise. **c**–**e** Measured spectral response of flat-top vs. DPSS-modulated pulses implementing ${\Bbb I}$ for different *k* and *NW*. Shading indicates the target band [0, *ω*_*B*_]. We choose *NW* = *k* + 1 for each *k*, to conservatively maintain spectral concentration of the DPSS-modulated pulses while matching the number of zero crossings in comparable flat-top protocols. Markers represent experimental measurements and solid lines show the analytic ${\cal F}_{{\mathrm{av}}}$. Arrows highlight out-of-band sensitivity due to harmonics of the flat-top control. We employed large modulation depths (*α* = 0.95 for DPSS-modulated pulses, *α* = 0.85 for flat-top pulses) to amplify these signals. Measurement sensitivity floor ~0.5%. As the pulse area and duration are fixed, the target bands within which the DPSS-modulated pulses are contained are broader than the main peaks of the flat-top pulses

We verify the spectral properties of DPSS-modulated qubit sensors by performing frequency-selective system identification to map out the effective spectral response of the driven qubit (Fig. 2a). A small single-frequency perturbation *β*_Ω_(*t*) = *α* cos(*ω*_sid_ *t* + *φ*) is added to the applied control envelope of the driving field, producing ${\mathrm{\Omega }}(t) \mapsto {\mathrm{\Omega }}(t)(1 + \beta _{\mathrm{\Omega }}(t))$. Since this results in *S*_Ω_(*ω*) ∝ *δ*(*ω* − *ω*_sid_), by scanning the tunable modulation frequency *ω*_sid_ and averaging over phase *φ* for fixed modulation depth *α*, we effectively reconstruct the filter transfer function of the control, *F*_Ω_(*ω*), through measurements of the average fidelity metric ${\cal F}_{{\mathrm{av}}}$. In this setting, we obtain ${\cal F}_{{\mathrm{av}}}$ directly from the projective measurements via $P\left( { \uparrow _z} \right) = 1 - {\cal F}_{{\mathrm{av}}} \approx 1 - {\mathrm{exp}}\left[ { - \pi ^{ - 1}{\int} {\mathrm{d}}\omega F_{\mathrm{\Omega }}(\omega )S_{\mathrm{\Omega }}(\omega )} \right]$, where we assume negligible dephasing. Experimental reconstruction of the qubits’ spectral response under DPSS-modulated pulses for *k* = 1 shows good agreement with the analytically calculated fidelity, in addition to the expected broadening as *NW* is increased (Fig. 2b).

Using the same technique, we can experimentally compare the frequency response of qubits driven by DPSS-modulated pulses to their flat-top counterparts, as shown in Fig. 2c–e. These experiments demonstrate the superior spectral concentration in the target band (shaded region); measurements on qubits subject to DPSS-modulated pulses show no detectable sensitivity to perturbations (given by *β*_Ω_(*t*)) outside of the target band, whereas flat-top pulses exhibit significant out-of-band harmonics (marked by arrows). Such sensor responses outside of the target band constitute a source of spectral leakage in sensing applications.

### Extending DPSS control capabilities

**Fig. 3:**
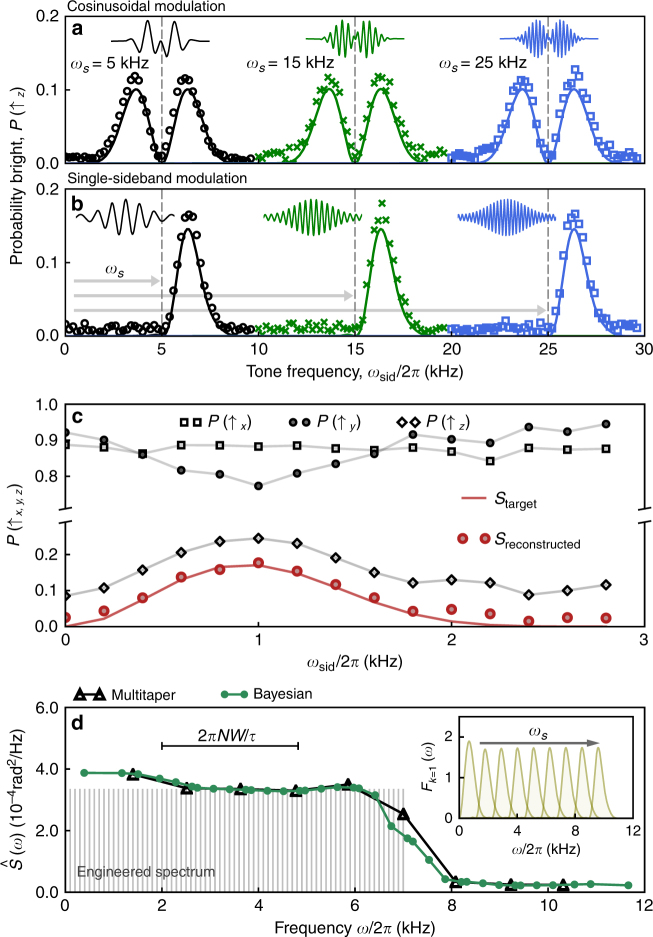
Sensing techniques and spectrum reconstruction with DPSS-modulated pulses. **a** Measurements (markers) and expected average fidelity ${\cal F}_{{\mathrm{av}}}$ (solid lines) for spectral response of band-shifted first-order DPSS filter functions with COS modulation. Modulation depth for system identification measurements was *α* = 0.5. The pulse shapes are shown as insets for each modulation frequency. **b** Band-shifted SSB modulated DPSS, using the same modulation frequencies as **a**. **c** Protocol for three axes measurements to reconstruct the amplitude filter function $\left( {{\cal S}_{{\mathrm{target}}}} \right)$ in the presence of white dephasing noise with a root-mean-square amplitude of 5Ω_*x*_, where Ω_*x*_ is the maximum Rabi frequency of the control. Rotations around the *x* and *y* axes allow preparation and readout along all three axes of the Bloch sphere. Projective measurements onto *x*, *y*, and *z* (black markers and lines), expected fidelity under amplitude noise only (red line), and fidelity reconstructed from the three projective measurements (red markers). System identification measurements were all taken with *α* = 0.75. **d** Reconstruction of an applied amplitude noise spectrum (shaded comb teeth) using adaptive and Bayesian multitaper estimation (see text). Inset shows all shifted *F*_Ω_(*ω*) for *k* = 1. Here, *ω*_*s*_ ranges from 1.4 to 10.0 kHz in steps of 1.1 kHz. Horizontal scale bar indicates target bandwidth of each measurement

In order to implement DPSS-modulated pulses for spectral reconstruction applications, we apply additional analog modulation techniques designed to shift the band center from zero to a user-defined frequency *ω*_*s*_^34^ (Methods). We employ two modulation protocols: cosinusoidal (COS) modulation shifts both positive and negative frequency components by *ω*_*s*_, while single-sideband (SSB) modulation shifts the band center by *ω*_*s*_ and suppresses either the positive or negative frequencies, thereby reducing the bandwidth by one half. Experiments using both techniques demonstrate maintenance of the critical spectral concentration of the DPSS-modulated pulses within the shifted bands (Fig. 3a, b). Further details are included in Supplementary Note 6.

Quantum sensing applications also require the ability to disambiguate changes in the measured operational fidelity due to target signals within a single quadrature, e.g., Ω*σ*_*x*_, from alternate “interfering” sources, which may be manifested similarly in projective measurements. For instance, the presence of a Hamiltonian term ∝*σ*_*z*_ during a driven operation ∝*σ*_*x*_ will reduce the measured fidelity of the driven operation in a manner similar to the presence of noise only proportional to the control^30^. Consequently, a single measurement is insufficient to determine which process is at play. To detect and compensate for such effects, we use tomographic reconstruction^35^, by preparing the qubit state along the three Cartesian directions, applying DPSS control, and performing independent sequential measurements in the corresponding bases (Fig. 3c). In our experiments, we simultaneously apply a target signal ∝*σ*_*x*_ as above, and an additional white dephasing term ∝*σ*_*z*_, which contributes to the sensor’s overall response in a way that obfuscates the measurement of the target. We then isolate the target signal’s contribution by combining three projective measurements as ${\cal S}$ ≡ (1 + *P*(↑_*x*_) − *P*(↑_*y*_) − *P*(↑_*z*_))/2, as derived in the Supplementary Note 3. Reconstructed values of ${\cal S}$ (red markers, Fig. 3c) reproduce the results expected without any *σ*_*z*_-terms well (red line), successfully correcting for a vertical offset that would otherwise bias a spectral estimate.

### Multitaper spectral reconstruction with DPSS controls

With demonstrations of the relevant band-limited properties of qubits subject to DPSS-modulated pulses as well as essential band-shifting techniques complete, we move on to demonstrate our spectral reconstruction capabilities. As a sample application, we reconstruct an engineered amplitude noise spectrum (Fig. 3d). We employ four different DPSS-modulated pulses with *k* = 1, 3, 5, 7, and *NW* = 7, band-shifted by SSB at nine different shift frequencies, *ω*_*s*_, each resulting in an individual estimate or “data taper”. The spacing of the modulation frequencies was chosen to be about 1/2 of the bandwidth of the filter functions, which yields measurements with sensitivity in overlapping bands. The various estimates are combined to produce a reconstruction of the target noise spectrum, which we accomplish this using two distinct techniques. While both are inspired by Thomson’s multitaper approach^20^, they also differ in important ways.

In its original form, the multitaper method aims to estimate the spectrum of a stationary Gaussian process from a finite set of discrete time samples. In this technique, DPSS waveforms are used to window the time–domain data in post-processing, producing a set of estimates of the spectrum in the target band. While each “single-taper” estimate is, in itself, an estimate of the spectrum *χ* ^2^-distributed about the true value, the key idea of multitaper estimation is to combine different estimates into a weighted-sum estimator with superior statistical properties. Thanks to the orthogonality of the DPSS, this procedure results in a *χ* ^2^-distribution with a greater number of degrees of freedom, ensuring consistency and increasing variance efficiency^20,21^. In order to offset the introduction of out-of-band bias from higher-order DPSS, the final estimate is determined through an adaptive weighting procedure, designed to favor the lowest-order estimates with the best spectral concentration in the band.

The first reconstruction technique we employ is closest in spirit to the original multitaper, with one crucial distinction; by applying DPSS amplitude modulation to the quantum sensor we are, in effect, windowing the noise process before any measurements are made. This stands in contrast to the manner in which classical multitaper estimates are determined by post-processing a set of discrete time samples. Measured fidelities determine preliminary spectral estimates at the center of each band, which are then weighted according to Thomson’s adaptive procedure to obtain a final set of estimates. In our second approach, we combine the use of multiple DPSS tapers with Bayesian estimation techniques. Each band, corresponding to a shift frequency, *ω*_*s*_, is sub-divided into a set of smaller segments. For each band, solving a linear inversion determines the Bayesian maximum a posteriori estimate of the spectrum in each segment, which serves as a preliminary estimate. As each segment is contained in multiple bands, the preliminary estimates are weighted by their Fisher information to determine a final estimate of the spectrum in each segment. Additional details on both reconstruction methods are given in the Supplementary Notes 8 and 9.

In our experiments, the Bayesian reconstruction in Fig. 3d uses the multitaper as a prior, and offers slightly improved resolution of the high-frequency cutoff. Both procedures produce spectral estimates which quantitatively match the applied spectrum (within resolution limits), and accurately identify the presence of a high-frequency cutoff in the noise. While the Bayesian approach relies on linear inversion and is thus computationally less efficient and stable than the adaptive multitaper, the flexibility in the choice of the model spectrum to be used as a prior, as well as the in-band segmentation, allow for improved resolution and the possibility to handle complex (e.g., “mixed”, consisting of both smooth and line components) spectra, in principle. Advantages of Bayesian spectral estimation are further highlighted in^36^.

### Characterization of native system noise

**Fig. 4:**
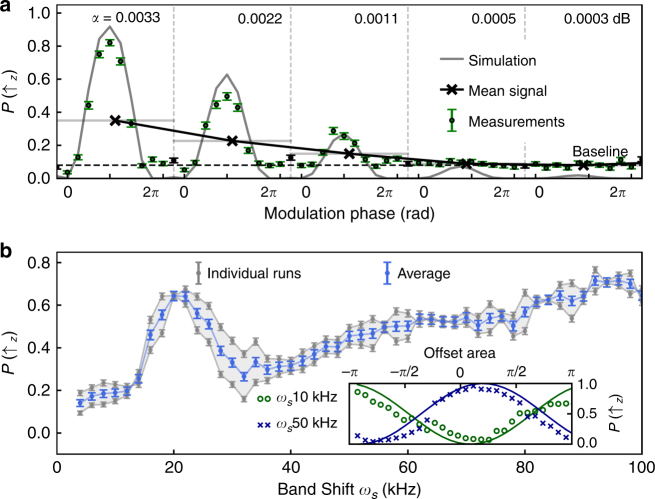
Sensing experiments of intrinsic amplitude noise using a single ion. **a** Sensitivity calibration using *k* = 0 and *ω*_sid_ = *ω*_*s*_, with *ω*_*s*_ = 10 kHz. Measurements are taken for linearly sampled phases from 0 to 2*π* with varying modulation depth and interleaved noiseless pulses to identify the fidelity limits of the experiments (“Baseline”). Each measurement is repeated 200 times; the resulting projection noise is captured by the error bars. The overall signal is represented as the average over all phase realizations. **b** Frequency-dependent amplitude instability probed with band-shifted *k* = 0 DPSS-modulated pulses. The measurement is repeated twice by scanning the *ω*_*s*_ first from 2–100 kHz and then from 100–2 kHz. The bandwidth of the DPSS filter (FWHM) is ~150 Hz. (inset) A small offset pulse is added at the beginning of the main protocol and its area is varied over the range [−*π*, *π*] to demonstrate the frequency-dependent pulse offset behavior. High-contrast fringes indicate quantum coherence is maintained

We conclude by using DPSS-modulated pulses to obtain frequency-resolved information about native noise and non-linearities in our control system at the end of the synthesis chain (which includes the vector signal generator, an amplifier, cabling, and a waveguide-to-coax converter). For this experiment, we use a single ion and perform DPSS-modulated pulses with *k* = 0, producing a net rotation equivalent to ~400*π* rotations, ideally enacting ${\Bbb I}$. We calibrate sensitivity to noise by first applying a single-frequency modulation at the shifted band center frequency, *ω*_sid_ = *ω*_*s*_, and averaging over phase (“x” markers, Fig. 4a). We compare this value against interleaved measurements conducted without applied noise to determine the minimum sensitivity achieving SNR ~1. These measurements demonstrate our ability to detect band-limited amplitude noise with modulation depth as low as ~0.001 dB. In measurements taken at different values of *ω*_*s*_, we observe a reproducible deviation from the ideal operation over much of the scan range, with a distinct feature around 20 kHz. We confirm that the measured signals are a manifestation of a frequency-dependent response in the synthesis chain rather than, e.g., extrinsic decoherence, by adding an initial small amplitude-offset pulse to a band-shifted DPSS-modulated pulse and scanning over the magnitude of that offset. Investigations into the source of this behavior are ongoing at the time this manuscript is prepared. Ultimately, this approach provides information that we believe is otherwise inaccessible via independent characterization of hardware components in our system.

## Discussion

The demonstrations presented here indicate that appropriately crafted quantum control protocols for qubit-based sensors have the ability to overcome significant technical limitations in contemporary quantum sensing experiments. These protocols can be applied to any qubit-based sensor in which arbitrary phase and amplitude modulation of the driving field is possible and spectral concentration is desired. It is noteworthy that by reducing the need to account for high-frequency harmonics in the Fourier response of the modulation pattern, the relatively simple and computationally efficient adaptive multitaper approach to spectrum reconstruction performs similarly to the more complex Bayesian estimation procedure under the conditions we tested. While a full comparative study of both single- and multitaper spectral reconstruction techniques using flat-top vs. DPSS-modulated pulses is beyond our current scope, preliminary simulations reinforce the utility of Slepian filters in mitigating leakage-induced artifacts in reconstruction. A detailed analysis will be the subject of an upcoming manuscript, along with developing mathematical bounds for spectral leakage and performing a quantitative assessment of the impact of leakage as a function of the target spectrum. Future experiments will also involve the extension of DPSS-modulated control to sensing of additive dephasing noise and multi-qubit settings, in order to provide an expanded toolkit of band-limited controls for quantum sensors.

## Methods

### Analog modulation techniques

Carrier waves are commonly modulated in radio-engineering to multiplex signals within a certain frequency band. We use the same approach to shift the sensitivity of our control pulses in the frequency domain. We employ two of these techniques, COS modulation (also commonly known as amplitude modulation), and single-sideband modulation. By multiplying the time–domain control pulses with a cosine function, so that ${\mathrm{\Omega }}_{{\mathrm{mod}},n}^{{\mathrm{COS}}} \equiv v_n^{(k)}(N,W){\mathrm{cos}}(n\omega _s{\mathrm{\Delta }}t)$, we convolve the original transfer function with two delta function at ±*ω*_*s*_. In a standard Fourier transform, the positive frequency component is reflected about the *y* axis. The negative frequency component becomes visible when *ω*_*s*_ is greater than the bandwidth of the pulse such that one copy of the positive and negative sidebands appear in the positive frequency domain. To recover the original appearance of the DPSS filter functions, we may alternatively use single-sideband modulation. In this case, the filter will either be at a higher or lower frequency than *ω*_*s*_, depending on the sign in ${\mathrm{\Omega }}_{{\mathrm{mod}},n}^{{\mathrm{SSB}}} \equiv v_n^{(k)}(N,W){\mathrm{cos}}(n\omega _s{\mathrm{\Delta }}t)$ ± ${\cal H}\left[ {v_n^{(k)}(N,W)} \right]{\mathrm{sin}}(n\omega _s{\mathrm{\Delta }}t)$. We pre-calculate the waveform numerically using the Hilbert transform, ${\cal H}\left[ {v_n^{(k)}(N,W)} \right]$, and apply it directly from our microwave source.

### Weak-noise limit

The regime in which the first-order fidelity approximation we employ is valid is the weak-noise limit. This requires that the smallness parameter, as defined in refs. ^28,30^, $\xi = \left[ {{\int}_0^\tau \mathrm{d}t\left\| {\beta (t)} \right\|} \right]^{1/2} < 1$. For the case of system identification, where *β*_Ω_(*t*) = *α* cos(*ω*_sid_ *t*), as well as for pure amplitude noise, *β*(*t*) = *β*_Ω_(*t*)Ω(*t*), we calculate $\xi = \alpha A{\mathrm{/}}2\sqrt 2$, where *A* is the pulse area. For pulses where *A* = *π*, the upper bound for the weak-noise limit is at *α* ≈ 0.9.

### Data availability

Data used in figures and computer scripts used to produce DPSS-modulated pulses and perform the spectral reconstruction are available at https://github.com/qcl-sydney/research-supplements.

## Electronic supplementary material

Supplementary Information
