# Supplementary material for: Application of optimal band-limited control protocols to quantum noise sensing
Source: Nat Commun. 2017 Dec 19;8:2189. doi: 10.1038/s41467-017-02298-2 (PMC5736660; doi:10.1038/s41467-017-02298-2)
Supplement: Supplementary file 1 — Supplementary Information [file 41467_2017_2298_MOESM1_ESM.pdf]

Supplementary Note 1 - Trapped ^{171}Yb^{+} ion qubits

The experimental systems considered in this work consist either of an ensemble of trapped ^{171}Yb^{+} ions or, for the intrinsic noise measurements, of a single trapped ion of the same species. Both configurations use a linear Paul trap enclosed in an ultra-high vacuum chamber. The ions are Doppler-cooled on the $^2\mathrm{S}_{1/2}$ to $^2\mathrm{P}_{1/2}$ transition using a 369 nm laser and additional repump lasers to depopulate undesired states. Qubits are realized by the magnetic dipole transition in the ground state hyperfine splitting between the states $|1\rangle \equiv {}^1\mathrm{S}_{1/2}\,|F=1, m_\mathrm{F}=0\rangle$ and $|0\rangle \equiv {}^1\mathrm{S}_{1/2}\,|F=0, m_\mathrm{F}=0\rangle$ with a transition frequency of approximately 12.6 GHz. In the Bloch sphere picture, these two states correspond to the eigenstates of the σ_z operator. State preparation and readout in z is accomplished optically via the $^2\mathrm{S}_{1/2}$ to $^2\mathrm{P}_{1/2}$ transition. Full details appear in [1, 2].

We drive the qubit transition using an low phase noise vector signal generator (Agilent E8267D) locked to an atomic caesium reference. The microwaves are amplified using a 18 dB amplifier (Microsemi AML618P1802) and delivered to the ions using two configurations for the different ion trap setups. For the ion ensemble, the amplifier output is routed through a conical microwave horn with a dielectric lens (Flann Microwave CL320-4901). For the single-ion experiments, we use a co-ax to waveguide converter (Flann Microwave 17094-SF40) which is butt-coupled to an optical viewport on the vacuum assembly. To implement arbitrary control protocols, we modulate the driving field through the inbuilt I/Q modulator, where we set our frame of reference such that pulses modulated by the I-component only implement rotations about x, and those modulated with the Q-component drive rotations about the y axis. Rotations about z, as performed in the experiments shown in Fig. 3, are implemented through frequency shifts of the carrier applied for a fixed amount of time. The control shapes presented in the main text are directly synthesised as either I or Q waveforms, which we compute digitally and which are passed through an intermediate digital-to-analog converter.

For experiments using engineered amplitude and (or) phase noise, we follow the protocol in [2].

Supplementary Note 2 - Control framework

The control-theoretic framework used in this work is described in detail in [3] and has been experimentally validated in [2, 4]. Here, we briefly reiterate the basic aspects necessary to support the experiments described in the main text.

A qubit subject to external control rotations about the x and y axes, implemented through modulation of the amplitude $\Omega(t)$ and phase of the driving field $\phi(t)$, evolves under the ideal control Hamiltonian (in units $\hbar = 1$)

$$H_\mathrm{c}(t) = \Omega(t)[\cos(\phi(t))\sigma_x + \sin(\phi(t))\sigma_y]/2, \qquad (1)$$

where a transformation to the interaction picture with respect to the qubit energy level splitting is understood. Any disturbance, either in the form of control-dependent amplitude and dephasing errors, or due to ambient environmental dephasing, leads to unwanted decoherence. We model the relevant noise sources as classical, in terms of a noise Hamiltonian

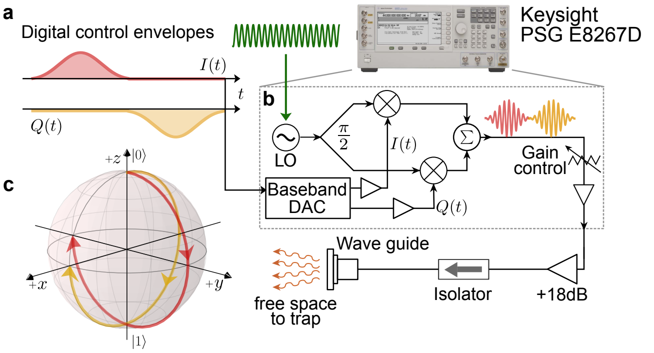

Supplementary Figure 1. Setup schematic of the single-ion experiment and implementation of qubit control. **a)** The control waveforms for I and Q are synthesized digitally and passed to the baseband DAC of vector signal generator. **b)** A simplified schematic of the key components of the I/Q modulation inside the vector signal generator. The output of the signal generator is amplified and a coax to waveguide converter creates free-space microwaves, which are routed into the trap. **c)** Bloch sphere representation of qubit control using I/Q modulation. I and Q implement perpendicular rotations around either the x or y axis, and state initialisation and readout are principally done by projecting onto z.

$$H_0(t) = \beta_z(t)\sigma_z + \beta_\Omega(t)H_\mathrm{c}(t), \qquad (2)$$

where $\beta_{z,\Omega}(t)$ are two independent zero-mean, stationary Gaussian random processes that represent multiplicative amplitude control noise and background dephasing noise, respectively. The full qubit evolution is thus determined by

$$H(t) = H_0(t) + H_\mathrm{c}(t) = \beta_z(t)\sigma_z + H_\mathrm{c}(t)[1 + \beta_\Omega(t)]. \quad (3)$$

It is convenient to work in a frame that co-rotates with the ideal control, making the noise processes the only source of rotation leading to deviations from the intended unitary evolution – a nontrivial gate Q in the most general case. In this frame, the effect of the noise on qubit dynamics may be isolated in terms of an "error propagator" [5] as

$$\tilde{U}(t) = \mathcal{T}_{+}\mathrm{exp}\left[-\mathrm{i}\int_0^t \mathrm{d}s\tilde{H}_0(s)\right], \; \tilde{H}_0(t) = U_\mathrm{c}(t)^\dagger H_0 U_\mathrm{c}(t), \quad (4)$$

where $U_\mathrm{c}(t)$ is the evolution operator generated by the ideal control Hamiltonian $H_\mathrm{c}(t)$ in Supplementary Equation (1). Over a duration τ, a gate $Q = U_c(\tau) \in \{\mathbb{I}, \sigma_x\}$ is implemented in our case, with the lab frame propagator being $U(\tau) = Q\tilde{U}(\tau)$. Since for a qubit $\tilde{U}(t) \in U(2)$, the latter may be equivalently expressed (up to a phase) as a rotation operator on the Bloch sphere:

$$\tilde{U}(t) = \exp[-\mathrm{i}\mathbf{a}(t)\cdot\boldsymbol{\sigma}], \qquad (5)$$

where the Pauli vector $\boldsymbol{\sigma} \equiv [\sigma_x, \sigma_y, \sigma_z]$, and the time-

dependent "error vector" $\mathbf{a}(t) \equiv [a_x(t), a_y(t), a_z(t)]$ captures the rotation error along the three coordinate axes. The error vector can be formally expressed in terms of a perturbative series expansion, $\mathbf{a}(t) = \sum_{n=1}^{\infty} \mathbf{a}^{(n)}(t)$, where each $\mathbf{a}^{(n)}(t)$ corresponds to the nth-order term in the Magnus expansion of $\tilde{U}(t)$. In the experimentally relevant regime, the noise is sufficiently weak to justify truncating the Magnus expansion to the leading (first) order, yielding $\mathbf{a}(t) \approx \mathbf{a}^{(1)}(t)$, with $a_i^{(1)}(t) = \mathrm{Tr}[\sigma_i \int_0^t \mathrm{d}s \tilde{H}_0(s)]/2$.

For the case of control generated by amplitude modulation along x as we use in the experiments, we may, without loss of generality, let $\phi(t) \equiv 0$ for all t in Supplementary Equation (1). This yields the control propagator $U_c(t) = \exp[-i\Theta(t)\sigma_x/2]$, where the net rotation angle is $\Theta(t) \equiv \int_0^t \mathrm{d}s\Omega(s)$. The leading contributions to the error vector components are then explicitly given by

$$a_x^{(1)}(t) = \int_0^t \mathrm{d}s\, \Omega(s)\beta_\Omega(s)/2, \quad (6)$$

$$a_y^{(1)}(t) = \int_0^t \mathrm{d}s \sin(\Theta(s))\beta_z(s), \quad (7)$$

$$a_z^{(1)}(t) = \int_0^t \mathrm{d}s \cos(\Theta(s))\beta_z(s). \quad (8)$$

Thus, amplitude noise is sensed by the x error-vector component, while dephasing noise enters both the y and the z components.

Supplementary Note 3 - Experimental measurables

To quantify the impact of noise in a way that is independent of a particular initial state, we consider the gate (or process) fidelity, averaged over all realizations of the noise Hamiltonian $H_0(t)$. This may be written as

$$\mathcal{F}_{\text{av}} = \frac{1}{4}\langle|\mathrm{Tr}(\tilde{U})|^2\rangle = \frac{1}{2}\Big(1 + \langle\cos(2|\mathbf{a}(t)|)\rangle\Big), \quad (9)$$

where $|\mathbf{a}(t)| = (\mathbf{a}(t)\cdot\mathbf{a}(t))^{1/2}$ is the length of the error vector.

To relate to experimentally measurable quantities, we consider projective measurements along z. If the qubit is prepared in the $|\uparrow_z\rangle$ ($= |0\rangle$, dark) eigenstate, and the target gate $Q = \mathbb{I}$, the survival probability (or state fidelity) in the state $|\uparrow_z\rangle$ after evolution under $U(t) = \tilde{U}(t)$ is found to be

$$\begin{aligned} P(\uparrow_z) &= \langle|\langle\uparrow_z|\tilde{U}(t)|\uparrow_z\rangle|^2\rangle \\ &= \Big\langle \frac{1}{2} + \frac{[a_z(t)]^2 + ([a_x(t)]^2 + [a_y(t)]^2)\cos(2|\mathbf{a}(t)|)}{2|\mathbf{a}(t)|^2} \Big\rangle \\ &\approx 1 - \langle[a_x^{(1)}(t)]^2\rangle - \langle[a_y^{(1)}(t)]^2\rangle, \end{aligned} \quad (10)$$

where we assumed that $|\mathbf{a}(t)|$ is sufficiently small to truncate the cosine at second order in its Taylor expansion.

For the experiments shown in the main text, excluding only Fig. 3c, any dephasing contributions to the overall signal are negligible ($\beta_z(t) \approx 0$), such that the first-order averaged fidelity is directly given by

$$\mathcal{F}_{\text{av}} = P(\uparrow_z) \approx 1 - \langle[a_x^{(1)}(t)]^2\rangle. \quad (11)$$

In general, from the above expressions for $\mathbf{a}^{(1)}(t)$, the reconstruction of amplitude noise requires access to $\langle[a_x^{(1)}(t)]^2\rangle$ only. For cases in which dephasing contributions are significant, the remaining components of $\mathbf{a}^{(1)}(t)$ can be reconstructed by using a three-axis measurement protocol. Similar to $P(\uparrow_z)$, the expected projections onto the x and y axes for a qubit initially prepared in $|\uparrow_x\rangle$ and $|\uparrow_y\rangle$, respectively, are given, to the leading order, by the expressions

$$P(\uparrow_x) \approx 1 - \langle[a_y^{(1)}(t)]^2\rangle - \langle[a_z^{(1)}(t)]^2\rangle, \quad (12)$$

$$P(\uparrow_y) \approx 1 - \langle[a_x^{(1)}(t)]^2\rangle - \langle[a_z^{(1)}(t)]^2\rangle. \quad (13)$$

Therefore, a combination of projective measurements in all three axes provides access to the individual components of the first-order error vector via

$$\langle[a_x^{(1)}(t)]^2\rangle \approx (1 + P(\uparrow_x) - P(\uparrow_y) - P(\uparrow_z))/2, \quad (14)$$

$$\langle[a_y^{(1)}(t)]^2\rangle \approx (1 + P(\uparrow_y) - P(\uparrow_x) - P(\uparrow_z))/2, \quad (15)$$

$$\langle[a_z^{(1)}(t)]^2\rangle \approx (1 + P(\uparrow_z) - P(\uparrow_x) - P(\uparrow_y))/2. \quad (16)$$

Experimentally, we may only measure in the qubit basis, therefore additional $\pi/2$ rotations must be implemented to measure the $P(\uparrow_x)$ and $P(\uparrow_y)$ projections. Supplementary Figure 2 shows how these measurements were implemented. Provided that all noise sources are sufficiently weak, Supplementary Equation (11) is then generalized to $\mathcal{F}_{\text{av}} \approx 1 - \langle|\mathbf{a}^{(1)}(t)|^2\rangle$. In the main text, we have used the short-hand notation $\mathcal{S} \equiv \langle[a_x^{(1)}(t)]^2\rangle$ in the context of discussing amplitude-noise sensing in the presence of non-negligible dephasing.

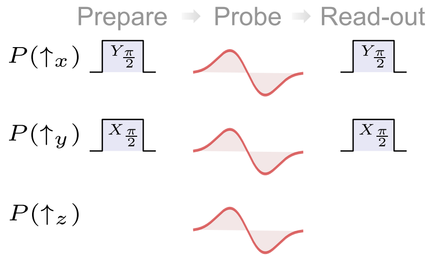

Supplementary Figure 2. Schematic representation of pulse sequences required to measure projections along the three axes, $P(\uparrow_x)$, $P(\uparrow_y)$, and $P(\uparrow_z)$, in order to achieve tomographic reconstruction. The ancillary $\pi/2$ pulses are implemented without the presence of any engineered noise.

As noted, the probabilities and error vector components defined above are given in the frame co-rotating with the ideal control. To access these quantities in the lab frame in the general case where $Q \neq \mathbb{I}$, the unitary evolution generated by the control and noise, $U(t)$, is followed by a fast rotation about the x axis, Q^{-1}, to effectively "undo" the rotation generated by the ideal control before the measurement. Since measurements are performed only in the z basis, in order to determine $P(\uparrow_x)$ and $P(\uparrow_y)$, an extra, fast, $\pi/2$ rotation is applied to the

qubit (about the y or x axis, respectively) to bring the state back to the z basis before the measurement (see also Supplementary Figure 2).

Supplementary Note 4 - Frequency domain filter functions

Under the assumptions for the noise processes $\beta_\Omega(t)$ and $\beta_z(t)$ mentioned above, the leading-order error vector has a spectral representation given by

$$\langle|\mathbf{a}^{(1)}(t)|^2\rangle = \frac{1}{2\pi}\sum_{i=\Omega,z}\int_{-\infty}^{+\infty}\frac{\mathrm{d}\omega}{\omega^2}S_i(\omega)F_i(\omega), \tag{17}$$

where $S_{\Omega,z}(\omega)$ are the power spectral densities of the dephasing and amplitude noise, and $F_{\Omega,z}(\omega)$ are filter-transfer functions of the applied control. Amplitude filter functions are the focus of this work, though a detailed treatment of both amplitude and dephasing filter functions can be found in [3, 4], with further generalization to a fully quantum setting in [6].

Since, for driven operations about the x axis with amplitude-modulated control envelopes, $a_x^{(1)}(t)$ depends only on the amplitude noise and filter function, we have

$$\langle|a_x^{(1)}(t)|^2\rangle = \frac{1}{2\pi}\int_{-\infty}^{\infty}\frac{\mathrm{d}\omega}{\omega^2}S_\Omega(\omega)F_\Omega(\omega). \tag{18}$$

In particular, let the control envelope be piecewise-constant, consisting of N intervals of duration Δt, with

$$\Omega(t) \equiv \Omega_n \quad \text{for} \quad n\Delta t \le t < (n+1)\Delta t, \tag{19}$$

and $n \in \{0,\dots,N-1\}$. Then the filter takes the form

$$\begin{aligned} F_\Omega(\omega) &= \omega^2\left|\int_0^t \mathrm{d}s e^{-i\omega s}\Omega(s)/2\right|^2 \\ &= \sin^2\!\left(\frac{\omega\Delta t}{2}\right)\left|\sum_{n=0}^{N-1}\Omega_n e^{i\omega[n-(N-1)/2]\Delta t}\right|^2. \end{aligned} \tag{20}$$

Supplementary Note 5 - Experimental DPSS control envelopes

For applications in sensing, we are interested in narrow-band filters for amplitude noise, where spectral weight or "leakage" outside the target band is suppressed. To generate such filters, we employ the discrete prolate spheroidal sequences (DPSS) or Slepians – namely, a family of finite, discrete-time sequences whose discrete-time Fourier transforms (DTFTs) are localized on a fixed frequency interval within the principal domain bounded by the Nyquist frequency, $\omega_N = \pi/\Delta t$ [7, 8]. For $k, n \in \{0,\dots,N-1\}$ and bandwidth parameter $W \in (0,1/2)$, the kth-order Slepian $\{v_n^{(k)}(N,W)\}$ is a real-valued sequence of length N satisfying the eigenvalue equation also given in the main text:

$$\sum_{m=0}^{N-1}\frac{\sin 2\pi W(n-m)}{\pi(n-m)}v_m^{(k)}(N,W) = \lambda_k(N,W)\,v_n^{(k)}(N,W). \tag{21}$$

The order of the Slepian is determined by the size of its eigenvalue with $1 > \lambda_0(N,W) > \ldots > \lambda_{N-1}(N,W) > 0$. The DTFT of $\{v_n^{(k)}(N,W)\}$ is the real-valued discrete prolate spheroidal wave-function (DPSWF) of order k,

$$U^{(k)}(N,W;\omega) = \epsilon_k\sum_{n=0}^{N-1}v_n^{(k)}(N,W)e^{i\omega[n-(N-1)/2]\Delta t}, \tag{22}$$

where $\epsilon_k = 1(i)$ for even (odd) k, and Δt and ω denote the sampling interval and angular frequency, respectively. The spectral concentration of $U^{(k)}(N,W;\omega)$ in the target frequency band $[-\omega_B,\omega_B] = [-2\pi W/\Delta t, 2\pi W/\Delta t]$, defined as the ratio of power in the target band to power in the principal domain $[-\omega_N,\omega_N]$, is then

$$\frac{\int_{-\omega_B}^{\omega_B}\mathrm{d}\omega\, U^{(k)}(N,W;\omega)^2}{\int_{-\omega_N}^{\omega_N}\mathrm{d}\omega\, U^{(k)}(N,W;\omega)^2} = \lambda_k(N,W), \tag{23}$$

and provably optimal amongst all sequences with same time and bandwidth parameters. By extending $\{v_n^{(k)}(N,W)\}$ to an infinite DPSS sequence with $n \in \mathbb{Z}$, concentration properties may be equivalently characterized in the time-domain as

$$\frac{\sum_{n=0}^{N-1}v_n^{(k)}(N,W)^2}{\sum_{n=-\infty}^{\infty}v_n^{(k)}(N,W)^2} = \lambda_k(N,W). \tag{24}$$

The first $\lfloor NW\rfloor$ DPSWFs, for which $\lambda_k(N,W)$ is close to 1, are the most spectrally concentrated, with the 0th-order DPSWF having the maximal spectral concentration over all DTFTs of finite, discrete-time sequences. Supplementary Table I contains the values of $\lambda_k(N,W)$ for $k \in \{0,10\}$ up to NW values of 5.

In order to take advantage of the spectral concentration of the DPSWFs, we use the piecewise-constant amplitude modulation in Supplementary Equation (19) with $\Omega_n = v_n^{(k)}(N,W)$. From Supplementary Equation (20), this creates the filter function

$$F_\Omega^{\mathrm{DPSS}}(\omega) = \sin^2\!\left(\frac{\omega\Delta t}{2}\right)U^{(k)}(N,W;\omega)^2, \tag{25}$$

spectrally concentrated in the band $[-\omega_B,\omega_B]$. The sinusoidal term appearing in $F_\Omega(\omega)$ is a consequence of the finite time duration Δt. Because this term enters the expression for $\langle|a_x^{(1)}(t)|^2\rangle$ in Supplementary Equation (18) as $\sin^2(\omega\Delta t/2)/\omega^2$, approaching $\Delta t^2/4$ for small ω, the spectral concentration of $U^{(k)}(N,W;\omega)$ is largely retained in this limit.

	$NW=1$	2	3	4	5
$k=0$	0.98105	0.99994	1.00000	1.00000	1.00000
1	0.74962	0.99756	1.00000	1.00000	1.00000
2	0.24359	0.95939	0.99972	1.00000	1.00000
3	0.02465	0.72176	0.99492	0.99997	1.00000
4	0.00107	0.27466	0.94615	0.99941	1.00000
5	$--$	0.04301	0.70782	0.99251	0.99993
6	–	0.00348	0.28978	0.93667	0.99907
7	–	$--$	0.05522	0.69885	0.99035
8	–	–	0.00593	0.29936	0.92932
9	–	–	$--$	0.06423	0.69236
10	–	–	$--$	0.00819	0.30622

Supplementary Table I. Scaling behavior of the eigenvalues $\lambda_k(N,W)$, i.e., spectral concentration of the Slepian wavefunctions, with different NW products for orders up to $k=2NW$. The values have been rounded to five decimal points. Numeric values smaller than 10^{-3} are denoted by –. In the experiments we generally restrict the applied orders to $k=NW-1$ (as highlighted in the table).

Supplementary Note 6 - Band-shifting protocols

By construction, the above DPSS-modulated filter $F_\Omega^{\text{DPSS}}(\omega)$ is spectrally concentrated with a pass-band centred at $\omega=0$. However, sensing applications require greater control over the central frequency of this band. In order to shift the frequency of the pass-band, we employ two modulation strategies. In co-sinusoidal modulation (COS), the amplitude modulation in Supplementary Equation (19) takes the form

$$\Omega_n^{\text{COS}} = v_n^{(k)}(N,W)\cos(n\omega_s\Delta t),$$

with band-shift frequency $\omega_s \geq 0$. For $\omega_s > 0$, this has the effect of shifting the original band by $\pm\omega_s$, producing a filter spectrally concentrated in two bands, $[-\omega_B \pm \omega_s, \omega_B \pm \omega_s]$. Due to symmetry of the spectrum and filter functions about $\omega=0$, we can equivalently view this filter as being spectrally concentrated solely in the band shifted by ω_s. The second approach is based on single-sideband modulation (SSB), a common technique in radio communication. For SSB, the amplitude modulation in Supplementary Equation (19) is given by

$$\Omega_n^{\text{SSB}} = v_n^{(k)}(N,W)\cos(n\omega_s\Delta t) - \hat{v}_n^{(k)}(N,W)\sin(n\omega_s\Delta t),$$

where $\{\hat{v}_n^{(k)}(N,W)\} \equiv \mathcal{H}[v_n^{(k)}(N,W)]$ denotes the discrete Hilbert transform of $\{v_n^{(k)}(N,W)\}$. SSB halves the band, shifting the negative half by $-\omega_s$ and the positive half by ω_s, creating a filter concentrated in the bands $[-\omega_s-\omega_B, -\omega_s]$ and $[\omega_s, \omega_B+\omega_s]$. Similar to COS, we can treat this filter as being spectrally concentrated in the positive band corresponding to ω_s due to symmetry about $\omega=0$.

In what follows, we denote by $F_{k,\omega_s}(\omega)$ the amplitude filter function produced by amplitude modulation using the Slepian $\{v_n^{(k)}(N,W)\}$, combined with either COS or SSB at band-shift frequency ω_s. Depending on which scheme is used, this filter is spectrally concentrated in the band

$$I_{\omega_s} = \begin{cases} \big[-\omega_B+\omega_s,\, \omega_B+\omega_s\big], & \text{COS} \\ \big[\omega_s,\, \omega_B+\omega_s\big], & \text{SSB}. \end{cases} \quad (26)$$

The "dual band" shifted by $-\omega_s$ is

$$I_{-\omega_s} = \begin{cases} \big[-(\omega_B+\omega_s),\, \omega_B-\omega_s\big], & \text{COS} \\ \big[-(\omega_B+\omega_s), -\omega_s\big], & \text{SSB}. \end{cases} \quad (27)$$

The measured fidelity of a qubit prepared in $|\uparrow_z\rangle$ after evolution under amplitude noise and filtering by $F_{k,\omega_s}(\omega)$ will be denoted by $\mathcal{F}_{k,\omega_s}$. For negligible dephasing, this gives directly $1-\langle [a_x^{(1)}(t)]^2\rangle$, whereas if this is not the case, $1-\langle [a_x^{(1)}(t)]^2\rangle$ can still be obtained via the three-axis measurement strategy, as noted. For brevity, we still refer to this quantity as $\mathcal{F}_{k,\omega_s}$.

Supplementary Note 7 - Application to qubit frequency measurement

We compared DPSS and primitive pulses in the measurement of the qubit resonance frequency by applying a pulse of fixed form and duration and scanning the microwave frequency (Supplementary Figure 3). Spectroscopy of the qubit transition frequency using a primitive pulse shows significant sub-peaks around the qubit resonance frequency, outside of the target band, as expected based on the Fourier transform of a square pulse. By contrast DPSS-modulated pulses show an overall concentration of spectral weight around the qubit transition frequency, commensurate with their superior spectral concentration as elucidated in the main text. The central lobe of the DPSS-modulated pulse for $k=0$ is slightly broader than that for a square pulse of the same area (see for comparison main text, Fig. 2c-e), but shows a dramatic suppression of off-resonant contributions outside of the target band. Using a DPSS-modulated pulse with $k=2$ and the same overall duration and net rotation area, we recover a narrowed central lobe while also maintaining overall spectral concentration, as expected for the first $(NW-1)$ DPSS-modulated pulses. Here, the presence of additional structure arises from the higher-order DPSS waveform in use, but all falls within the target band (indicated by blue shading). This phenomenology is comparable to that observed in Fig. 2c-e of the main text, where higher order DPSS pulses can show complex structure in the filter function, but this structure remains inside of the target band.

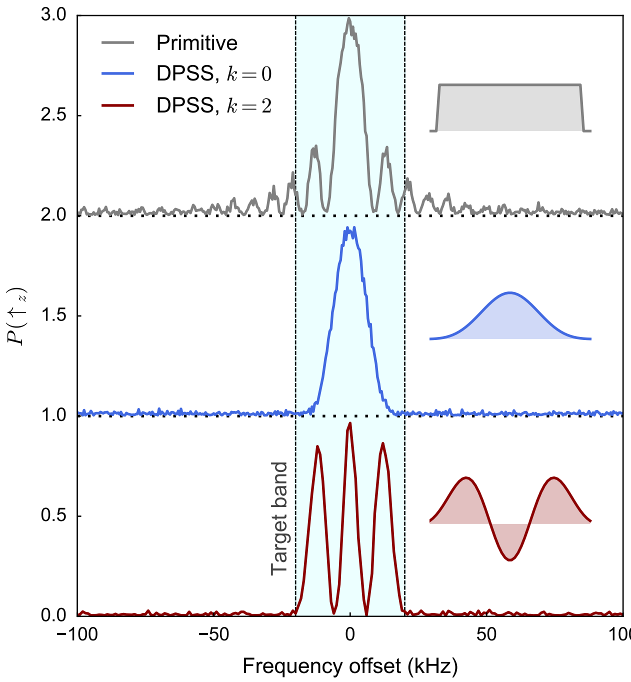

Supplementary Figure 3. Comparison between measurements of the qubit resonance frequency using primitive vs. DPSS-modulated pulses. Top measurement was taken with a flat-top profile with net area 3π, the middle measurement used a $k = 0$ DPSS-modulated pulse with net area 3π, whereas the lower measurement used a $k = 2$ DPSS-modulated pulse with a net area 1π. $NW = 3$ for both orders. Shaded region indicates target band.

Supplementary Note 8 - Spectral reconstruction via adaptive multitaper approach

Our first reconstruction method follows Thomson's original approach to multitaper spectral estimation [9], with the essential difference being that the Slepian window functions are applied "online", before the acquisition of experimental data. For a measured fidelity $\mathcal{F}_{k,\omega_s}$, the kth estimate is

$$\hat{S}^{\text{m}}_k(\omega_c) = \frac{1-\mathcal{F}_{k,\omega_s}}{A_{k,\omega_s}},\ \ A_{k,\omega_s} = \int_{I_{\omega_s}\cup I_{-\omega_s}} \frac{\text{d}\omega}{2\pi\omega^2} F_{k,\omega_s}(\omega). \tag{28}$$

The estimation frequency, $\omega_c \geq 0$, is the center of I_{ω_s}, that is,

$$\omega_c = \begin{cases} \omega_s, & \text{COS} \\ \omega_s + \omega_B/2, & \text{SSB}. \end{cases} \tag{29}$$

In order to ensure consistency of the estimator and to increase the variance efficiency, the estimates for each Slepian order are combined into a weighted average to produce a multitaper spectral estimate, namely,

$$\hat{S}^{\text{m}}(\omega_c) = \sum_k d_k(\omega_c)\hat{S}^{\text{m}}_k(\omega_c), \quad \sum_k d_k(\omega_c) = 1. \tag{30}$$

However, the inclusion of higher-order Slepians with less spectral concentration has the potential to bias the estimate. To compensate for this, Thomson introduced an adaptive weighting procedure to determine the $d_k(\omega_c)$, whereby estimates expected to introduce more bias are down-weighted.

To arrive at the final estimate in Supplementary Equation (30), we employ a variation of this procedure depending on two sources of bias. The estimate in Supplementary Equation (28) can be expressed as a sum of the true spectrum and the broad-band and local biases, namely,

$$\hat{S}^{\text{m}}_k(\omega_c) = S(\omega_c) + B^{\text{BB}}_{k,\omega_s} + B^{\text{LOC}}_{k,\omega_s}.$$

The broad-band bias, which quantifies error due to the spectral concentration of the filter outside I_{ω_s}, is given by

$$B^{\text{BB}}_{k,\omega_s} = \frac{1}{2\pi A_{k,\omega_s}}\Big[\int_{-\infty}^{\infty} \frac{\text{d}\omega}{\omega^2} F_{k,\omega_s}(\omega)\, S(\omega) - \tag{31}$$

$$\int_{I_{\omega_s}\cup I_{-\omega_s}} \frac{\text{d}\omega}{\omega^2} F_{k,\omega_s}(\omega)\, S(\omega)\Big]. \tag{32}$$

The local bias, which quantifies error due to the curvature of the spectrum within the band, takes instead the form

$$B^{\text{LOC}}_{k,\omega_s} = \frac{1}{\pi A_{k,\omega_s}}\Big[\int_{I_{\omega_s}} \frac{\text{d}\omega}{\omega^2} F_{k,\omega_s}(\omega) \sum_{p=1}^{\infty} \frac{S^{(p)}(\omega_c)(\omega-\omega_c)^p}{p!} \tag{33}$$

$$- \int_{I_{\omega_s}\cap I_{-\omega_s}} \frac{\text{d}\omega}{\omega^2} F_{k,\omega_s}(\omega) \sum_{p=1}^{\infty} \frac{S^{(p)}(\omega_c)(\omega-\omega_c)^p}{2p!}\Big]. \tag{34}$$

In our analysis, we truncate the Taylor expansions in this expression at the first order (linear) terms.

The first step in determining the $d_k(\omega_c)$ is solving for a set of unnormalized weighting coefficients, $\tilde{d}_k(\omega_c)$, that minimize the squared error, $E_k(\omega_c) = \big[S(\omega_c) - \tilde{d}_k(\omega_c)\hat{S}^{\text{m}}_k(\omega_c)\big]^2$. This can be carried out analytically, yielding

$$\tilde{d}_k(\omega_c) = \frac{S(\omega_c)}{[S(\omega_c) + B^{\text{BB}}_{k,\omega_s} + B^{\text{LOC}}_{k,\omega_s}]},$$

which depends on the unknown spectrum both explicitly and implicitly through the bias terms. In order to proceed, $S(\omega)$ is replaced by a prior estimate of the spectrum, $\hat{S}^{(0)}(\omega)$. In our analysis, $\hat{S}^{(0)}(\omega)$ was taken to be a linear interpolation of the set of estimates from the first-order Slepian in each band. The prior determines estimates for the broad-band and local biases, which are then substituted into the analytic formula to produce a first-order solution for the unnormalized weighting coefficients, $\tilde{d}^{(1)}_k(\omega_c)$. This is is the first step in a recursion. At iteration n, the unnormalized weighting coefficients are

$$\tilde{d}^{(n)}_k(\omega_c) = \frac{\hat{S}^{(n-1)}(\omega_c)}{\hat{S}^{(n-1)}(\omega_c) + \hat{B}^{\text{BB}\,(n-1)}_{k,\omega_s} + \hat{B}^{\text{LOC}\,(n-1)}_{k,\omega_s}}. \tag{35}$$

Next, the nth-order spectral estimate is determined from Eq. (30) by normalizing the $\tilde{d}_k^{(n)}(\omega_c)$,

$$\hat{S}^{(n)}(\omega_c) = \frac{\sum_k \tilde{d}_k^{(n)}(\omega_c)\hat{S}_k^{\mathrm{m}}(\omega_c)}{\sum_k \tilde{d}_k^{(n)}(\omega_c)}. \quad (36)$$

This process is repeated until the weighting parameters converge (after ~ 5 iterations in the multitaper reconstruction of Fig. 3d). A substantial advantage of the multitaper approach is that it is free of linear inversion, making it computationally efficient and numerically stable.

Supplementary Note 9 - Spectral reconstruction via Bayesian multitaper approach

For each band-shift frequency ω_s, the multitaper approach produces an estimate of the spectrum at the center of band I_{ω_s}. The achievable resolution is, thus, limited by the number of band-shift frequencies. For a finer sampling, we pursued a second technique capable of reconstructing the spectrum at multiple frequencies within each I_{ω_s}, using a Bayesian maximum a posteriori (MAP) estimate. This approach is particularly amenable to SSB-modulated Slepian filters, which tend to be more delocalized within I_{ω_s}, enabling us to infer information about the spectrum in different regions of the band.

The Bayesian procedure estimates the spectrum on a finer grid of "segments" within each band. Consider a set of band-shift frequencies $\omega_s = 0, \omega_0, \ldots, L\omega_0$. Each band $I_{\ell\omega_0}$, corresponding to modulation frequency $\ell\omega_0$, is divided into M segments of width $\Delta\omega$ along the positive frequency axis, $\mathrm{seg}_{\ell,1}, \ldots, \mathrm{seg}_{\ell,M}$, where the average value of the spectrum in $\mathrm{seg}_{\ell,m}$ is $\overline{S}_{\ell,m}$. In the first stage of the reconstruction, we estimate the average values of the spectrum in all segments and bands,

$$\overline{S} \equiv \big\{\overline{S}_{\ell,m} \big| \ell=1,\ldots,L;\, m=1,\ldots,M\big\}.$$

If the fidelity is measured for Slepians of order $k_1, \ldots, k_d$ at each band-shift frequency, $\overline{S}$ is estimated by the maximum of its posterior distribution given the experimental data,

$$P\big(\overline{S}|\{\mathcal{F}_{k_i,\ell\omega_0}\}\big) = \frac{\prod_{i=1}^d \prod_{\ell=1}^L P(\mathcal{F}_{k_i,\ell\omega_0}|\overline{S})\, P(\overline{S})}{\prod_{i=1}^d \prod_{\ell=1}^L P(\mathcal{F}_{k_i,\ell\omega_0})}. \quad (37)$$

In terms of $\overline{S}$, the true fidelity is approximately

$$\mathcal{F}^{(T)}_{k_i,\ell\omega_0} = 1 - \frac{\Delta\omega}{\pi}\sum_{m=1}^M \overline{F}_{k_i,\ell\omega_0,m}\,\overline{S}_{\ell,m},$$

where $\overline{F}_{k_i,\ell\omega_0,m}$ is the average value of $F_{k,\ell\omega_0}(\omega)/\omega^2$ in segment m. Because the primary source of error in the fidelity measurements is photon shot noise, the likelihood of measuring $\mathcal{F}_{k_i,\ell\omega_0}$ given $\overline{S}$ is Gaussian distributed about the true fidelity to good approximation, allowing us to write

$$P\big(\mathcal{F}_{k_i,\ell\omega_0}\big|\overline{S}\big) = \mathcal{N}\exp\Bigg[-\frac{(\mathcal{F}_{k_i,\ell\omega_0} - \mathcal{F}^{(T)}_{k_i,\ell\omega_0})^2}{2\sigma^2_{k_i,\ell\omega_0}}\Bigg]. \quad (38)$$

The prior distribution in Supplementary Equation (37) is taken to be Gaussian about a preliminary estimate $\hat{S}^{(0)}_{\ell,m}$, with uncertainty σ_0,

$$P\big(\overline{S}\big) = \mathcal{N}\prod_{\ell=1}^{L}\prod_{m=1}^{M}\exp\Bigg[-\frac{\big(\overline{S}_{\ell,m} - \hat{S}^{(0)}_{\ell,m}\big)^2}{2\sigma_0^2}\Bigg]. \quad (39)$$

The Bayesian reconstruction in Fig. 3d uses the multitaper estimate as a prior. The finer sampling of the spectrum enabled by the Bayesian procedure refines this initial estimate, improving the resolution of the high-frequency cutoff. If prior information about the spectrum is unavailable, taking $\sigma_0 \gg \max\{\hat{S}^{(0)}_{\ell,m}\}$ produces an effectively flat prior.

By maximizing Supplementary Equation (37) with respect to $\overline{S}$, we determine that the MAP estimates in band $I_{\ell\omega_0}$, denoted by $\{\hat{S}_{\ell,1}, \ldots, \hat{S}_{\ell,M}\}$ are the solutions of a linear system, $\vec{V} = R\vec{S}$, where $\vec{S} = \big(\hat{S}_{\ell,1}, \ldots, \hat{S}_{\ell,M}\big)^T$, $\vec{V}$ is an $M \times 1$ vector with elements

$$V_m = \sum_{i=1}^{d}\frac{\sigma_0^2\,\Delta\omega}{\pi\,\sigma^2_{k_i,\ell\omega_0}}\overline{F}_{k_i,\ell\omega_0,m}\,(1 - \mathcal{F}_{k_i,\ell\omega_0}) + \hat{S}^{(0)}_{\ell,m}, \quad (40)$$

and R is a $M \times M$ matrix with elements

$$R_{m,m'} = \sum_{i=1}^{d}\frac{\sigma_0^2\,\Delta\omega^2}{\pi^2\sigma^2_{k_i,\ell\omega_0}}\overline{F}_{k_i,\ell\omega_0,m}\overline{F}_{k_i,\ell\omega_0,m'} + \delta_{m,m'}. \quad (41)$$

Solving the linear system for $\ell = 1, \ldots, L$ produces an estimate of $\overline{S}$, the average value of the spectrum in all segments and bands. The conditioning of the linear inversion places a limit on M, the number of estimates per band. In general, the inversion will be well-conditioned if each filter has most of its spectral weight in a single segment.

The spacing between the band-shift frequencies, ω_0, is generally smaller than the width of the bands. This means that the bands will overlap and a segment $\mathrm{seg}_{\ell,m}$ associated with band $I_{\ell\omega_0}$ can be contained in one or more additional bands. In particular, if $\mathrm{seg}_{\ell,m}$ is contained in $I_{\ell'\omega_0}$ in addition to $I_{\ell\omega_0}$, the measured fidelities $\mathcal{F}_{k_1,\ell'\omega_0}, \ldots, \mathcal{F}_{k_d,\ell'\omega_0}$ contain information about $\overline{S}_{\ell,m}$. In the second stage of the Bayesian multitaper approach, we incorporate the information about $\overline{S}_{\ell,m}$ available in other bands in order to determine a final estimate $\hat{S}^{\mathrm{b}}_{\ell,m}$. Motivated by Thomson's high resolution expansion [9], we quantify the information about $\overline{S}_{\ell,m}$ contained in $\mathcal{F}_{k_1,\ell'\omega_0}, \ldots, \mathcal{F}_{k_d,\ell'\omega_0}$ by the classical Fisher information,

$\mathcal{I}_{\{\mathcal{F}_{k_i,\ell'\omega_0}\}}(\overline{S}_{\ell,m})$. From direction calculation,

$$\mathcal{I}_{\{\mathcal{F}_{k_i,\ell'\omega_0}\}}(\overline{S}_{\ell,m}) = \sum_{i=1}^{d} \left[\frac{1}{\pi\sigma_{k_i,\ell'\omega_0}} \int_{\mathrm{seg}_{\ell,m}} \frac{\mathrm{d}\omega}{\omega^2} F_{k_i,\ell'\omega_0}(\omega)\right]^2. \tag{42}$$

If $O(\mathrm{seg}_{\ell,m}) = \{(\ell',m') | \mathrm{seg}_{\ell,m} \cap \mathrm{seg}_{\ell',m'} \neq \emptyset\}$ contains all indices of the segments overlapping $\mathrm{seg}_{\ell,m}$, the final estimate of $\overline{S}_{\ell,m}$ is a weighted average of estimates in the overlapping segments, yielding

$$\hat{S}^{\mathrm{b}}_{\ell,m} = \frac{\sum_{(\ell',m')\in O(\mathrm{seg}_{\ell,m})} \mathcal{I}_{\{\mathcal{F}_{k_i,\ell'\omega_0}\}}(\overline{S}_{\ell,m}) \hat{S}_{\ell',m'}}{\sum_{(\ell',m')\in O(\mathrm{seg}_{\ell,m})} \mathcal{I}_{\{\mathcal{F}_{k_i,\ell'\omega_0}\}}(\overline{S}_{\ell,m})}. \tag{43}$$

The weight on each estimate increases with the information it carries about $\overline{S}_{\ell,m}$.

SUPPLEMENTARY REFERENCES

[1] S. Mavadia, V. Frey, J. Sastrawan, S. Dona, and M. J. Biercuk, "Prediction and real-time compensation of qubit decoherence via machine learning," *Nature Commun.*, vol. 8, p. 14106, (2017).

[2] Soare, A. *et al.*, "Experimental bath engineering for quantitative studies of quantum control," *Phys. Rev. A*, vol. 89, p. 042329, (2014).

[3] Green, T. J. and Sastrawan, J. and Uys, H. and Biercuk, M. J. , "Arbitrary quantum control of qubits in the presence of universal noise," *New J. Phys.*, vol. 15, p. 095004, (2013).

[4] Soare, A. *et al.*, "Experimental noise filtering by quantum control," *Nature Phys.*, vol. 10, pp. 825–829, 2014.

[5] K. Khodjasteh and L. Viola, "Dynamically error-corrected gates for universal quantum computation," *Phys. Rev. Lett.*, vol. 102, p. 080501, (2009).

[6] Paz-Silva, G. A. and Viola, L., "General transfer-function approach to noise filtering in open-loop quantum control," *Phys. Rev. Lett.*, vol. 113, p. 250501, (2014).

[7] Slepian, D. and Pollak, H. O., "Prolate spheroidal wave functions, fourier analysis, and uncertainty -I," *Bell Syst. Tech. J.*, vol. 40, pp. 43–63, (1961).

[8] Landau, H. J. and Pollak, H. O., "Prolate spheroidal wave functions, fourier analysis, and uncertainty -II," *Bell Labs Technical Journal*, vol. 40, pp. 65–84, (1961).

[9] Thomson, D. J., "Spectrum estimation and harmonic analysis," *Proceedings of the IEEE*, vol. 70, pp. 1055–1096, (1982).
